# Dual ACL Ganglion Cysts: Significance of Detailed Arthroscopy

**DOI:** 10.1155/2014/236902

**Published:** 2014-10-08

**Authors:** Samarth Mittal, Amit Singla, H. L. Nag, Sanjay Meena, Ramprakash Lohiya, Abhinav Agarwal

**Affiliations:** ^1^Department of Orthopaedics, All India Institute of Medical Sciences, New Delhi, India; ^2^SPMC, Bikaner, Rajasthan, India

## Abstract

Intra-articular ganglion cysts of the knee joint are rare and most frequently are an incidental finding on MRI and arthroscopy. Most of the previous studies have reported a single ganglion cyst in the knee. There have been previous reports of more than one cyst in the same knee but not in the same structure within the knee. We are reporting a case of dual ACL (anterior cruciate ligament) ganglion cysts one of which was missed on radiological examination but later detected during arthroscopy. To the best of our knowledge, no such case has been reported in the indexed English literature till date.

## 1. Introduction

Ganglion cysts related to anterior cruciate ligament (ACL) are rare. Reported incidence varies from 0.29% [1] to 1.3% [2] on MRI and 0.54% [3] on arthroscopic examination of knee joint. The most common site for cystic lesions inside the knee joint is ACL, PCL, and the menisci in descending order of frequency [4]. The purpose of this report is to present a case with two intra-articular ganglion cysts in the knee joint originating from the anterior cruciate ligament. To the best of our knowledge, no such case has been reported in the indexed English literature till date. The patient, with history of painful restriction in movement in flexion and terminal extension, was diagnosed with posterior ACL ganglion cyst on MRI but on arthroscopy it was found out that the patient has two cysts arising from ACL. These were successfully removed and confirmed to be ganglion cysts on histopathological examination. The symptoms resolved after the surgery. Such finding of two ganglion cysts in a single ACL is rare; hence the operating surgeon should be aware of such situation in order to decrease the chances of missing one. Also a detailed diagnostic arthroscopic evaluation must be conducted in all cases undergoing arthroscopic surgery so as to detect the lesions which can be occasionally missed on a MRI examination as was seen in our case.

## 2. Case Report

A 28-year-old female presented with pain in her right knee joint for the past 18 months. She was a housewife and had a history of twisting injury to the right knee two years prior to presentation. At presentation, she had pain in the right knee, both beyond 120-degree flexion and in terminal extension. On clinical examination, there was mild wasting of the quadriceps muscle. Range of movement was full but both terminal knee flexion and extension were painful. Clinical tests for cruciate knee ligaments and meniscal injuries were negative.

Magnetic resonance imaging (MRI) was reported as large lobulated multiloculated cystic lesion near the posterior aspect of the femoral attachment of ACL suggesting encysted synovial collection or cystic synovial neoplasm.

**Figure 1:**
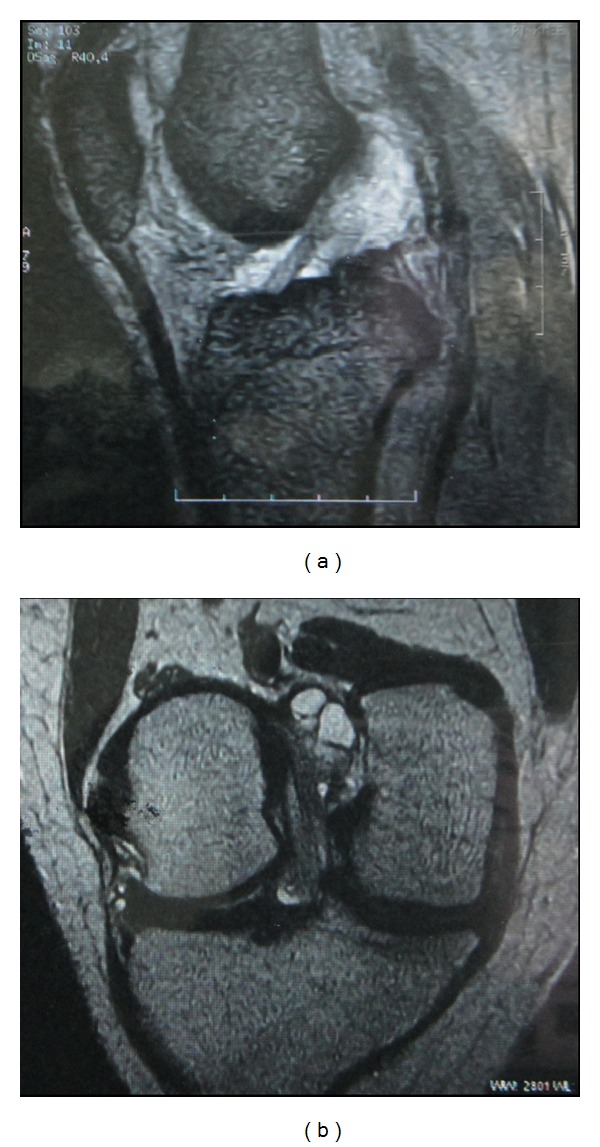
MRI images of ACL ganglion cysts.

**Figure 2:**
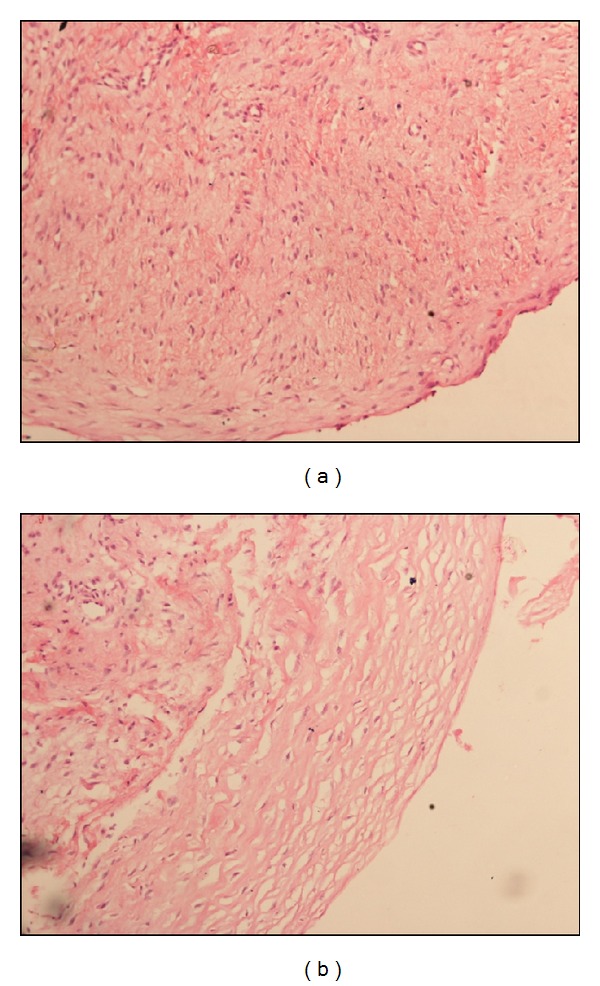
Light microscopy (10x) images of wall of ganglion cysts.

During arthroscopic evaluation, the cyst was visualised on the posterior extrasubstance aspect of ACL near femoral attachment measuring approximately 15 mm by 12 mm in diameter. A second cyst, not connected with the first, was noted anterior to ACL near the tibial attachment measuring around 5 mm by 3 mm in diameter which was not reported in the MRI. However, after surgery, a review of the same MRI also showed the presence of the anterior ACL cyst (Figures 1(a) and 1(b)). The cysts were thin walled, transparent, freely mobile, and well demarcated and their origin from ACL was clearly visualized. There were no adhesions or signs of inflammation surrounding them. The medial and lateral menisci were normal, ACL, PCL were intact, and the rest of the joint was normal. The cysts were punctured and their walls broken by probing. Clear gelatinous fluid was seen exuding from the cysts when they were punctured. Arthroscopy resector was used to resect the majority of the cyst wall and the same was sent for histopathological examination. ACL was confirmed to be intact after the procedure. Postoperatively patient was mobilized in the hospital and there was no pain during terminal movements on the day of surgery itself. Biopsy of the lesion was compatible with that of the ganglion cyst (Figures 2(a) and 2(b)).

**Figure 3:**
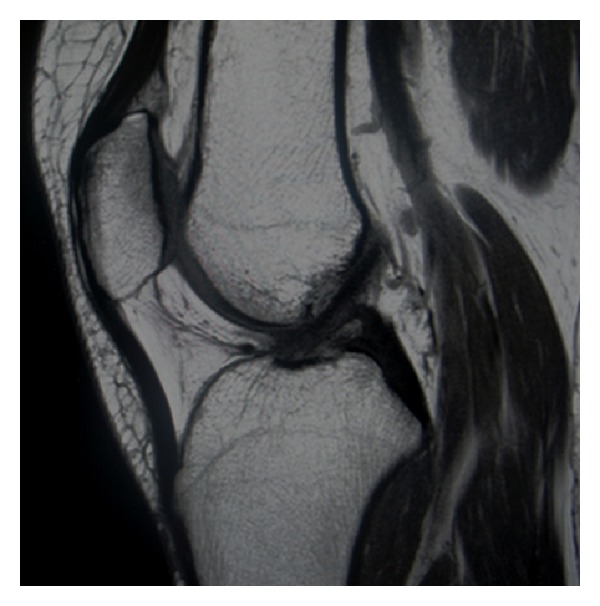
MRI images of patient at final follow-up showing absence of any ganglion cyst.

The patient was followed up in orthopaedic outpatient department (OPD) regularly. At the final 2-year postoperative follow-up, the patient did not have any complaints and the entire range of joint motion was painless with a repeat MRI not showing presence of either of the ganglion cysts (Figure 3).

## 3. Discussion

Ganglion is a cystic swelling which is often seen originating near tendons or joints [4]. It occurs most commonly on dorsum of the hand [4]. Nearly two-thirds of all knee ganglion cysts originate from the tibial insertion of ACL [4]. Multiple ganglion cysts have been reported in the past but from different origins like one from ACL and another from lateral meniscus [5]; in our case both the cysts were arising from anterior cruciate ligament. They appear uni- or multilocular and are usually found singular in each knee. According to published literature, history of trauma is present in almost 38% to 67% of patients prior to development of symptoms [3]. Pain is the most common symptom. They are also frequently associated with recurrent effusions or locking in extremes of flexion or extension, mimicking meniscal injuries [6]. Location of the cyst plays a major role in the limitation of knee joint movement with cysts. Limitation of extension is noted in cysts anterior to the cruciate ligament, whereas flexion of the knee is mostly affected in cysts posterior to cruciate ligament [7, 8]. Krudwig et al. [9] reported 85 cases of intra-articular ganglion cysts, of which 9 were symptomatic; all the 9 symptomatic patients had no history of trauma. The definite history of trauma with our patient is a significant finding. Such cystic lesions are best visualized by MRI which will not only show the position and the dimensions but also differentiate it from malignant lesions and other joint abnormalities [1]. However, unlike in other locations such as the ankle joint or dorsal-side wrist, recurrence is rare after arthroscopic surgical treatment for ganglion cysts of the knee [10]. The exact reason for this phenomenon is not known. Brown and Dandy [3] reported a 95% success rate after arthroscopic excision of the cysts with no recurrence in any of the patients.

## References

[1] Huang G-S, Lee C-H, Chan WP (2002). Ganglion cysts of the cruciate ligaments. *Acta Radiologica*.

[2] Kim MG, Kim BH, Choi J-A (2001). Intra-articular ganglion cysts of the knee: clinical and MR imaging features. *European Radiology*.

[3] Brown MF, Dandy DJ (1990). Intra-articular ganglia in the knee. *Arthroscopy*.

[4] Dinakar B, Khan T, Kumar AC (2005). Ganglion cyst of the anterior cruciate ligament: a case report. *Journal of Orthopaedic Surgery*.

[5] Wang C-J (2002). Multiple ganglion cysts of the knee. *Arthroscopy*.

[6] Kang C-N, Kim D-W, Kim D-J, Kim S-J (1999). Intra-articular ganglion cysts of the knee. *Arthroscopy*.

[7] Höcker K, Jagenbrein G, Schwarz N, Ritschl P (1996). Painful functional impairment of the knee joint caused by an ACL-based ganglion cyst. *Injury*.

[8] Johnson WL, Corzatt RD (1993). Ganglion cyst of the anterior cruciate ligament: a case report of an unusual cause of mechanical knee symptoms. *The American Journal of Sports Medicine*.

[9] Krudwig WK, Schulte K-K, Heinemann C (2004). Intra-articular ganglion cysts of the knee joint: a report of 85 cases and review of the literature. *Knee Surgery, Sports Traumatology, Arthroscopy*.

[10] Andrikoula SI, Vasiliadis HS, Tokis AV, Kosta P, Batistatou A, Georgoulis AD (2007). Intra-articular ganglia of the knee joint associated with the anterior cruciate ligament: a report of 4 cases in 3 patients. *Arthroscopy*.

